# Translation into Russian of: “Changes to publication requirements made at the XVIII International Botanical Congress in Melbourne – what does e-publication mean for you?” Translated by Irina V. Belyaeva and Maria S. Vorontsova Изменения требований к обнародованию, принятые на XVIII Международном Ботаническом Конгрессе в Мельбурне – что означает электронное обнародование?

**DOI:** 10.3897/phytokeys.6.2001

**Published:** 2011-09-14

**Authors:** Sandra Knapp, John McNeill, Nicholas J. Turland

**Affiliations:** 1 Department of Botany, The Natural History Museum, Cromwell Road, London SW7 5BD, UK The Natural History Museum London United Kingdom; 2 Royal Botanic Garden, Edinburgh, 20A Inverleith Row, Edinburgh EH3 5LR, UK Royal Botanic Garden Edinburgh United Kingdom; 3 Missouri Botanical Garden, PO Box 299, St Louis, MO 63166-0299, USA Missouri Botanical Garden St. Louis United States of America

## Abstract

Решения по изменениям *Международного Кодекса Ботанической Номенклатуры *принимаются каждые 6 лет на заседании Номенклатурной Секции Международного Ботанического Конгресса (МБК). XVIII МБК состоялся в Мельбурне, Австралия; Номенклатурная Секция заседала 18-22 июля 2011 г., и ее решения были утверждены Конгрессом на пленарном заседании 30 июля. В ходе этого заседания было сделано несколько важных изменений в *Кодексе*, которые затрагивают обнародование новых названий.Два из этих изменений вступят в действие с 1 января 2012 г., несколькими месяцами раньше, чем *Мельнбурнский Кодекс* будет опубликован. Электронные материалы, опубликованные онлайн в формате PDF (Portable Document Format) с Mеждународным Стандартным Серийным Номером ISSN (International Standard Serial Number) или с Международным Стандартным Книжным Номером ISBN (International Standard Book Number) будут считаться эффективно обнародованными, а требование наличия латинского описания или диагноза при обнародовании новых названий будет заменено требованием наличия описания на латинском или английском языке. В дополнение к этому, чтобы быть эффективно и действительно обнародованными, с 1 января 2013 г. новые названия организмов, считающихся грибами, должны цитировать в протологе (все, что связано с названием при его действительной публикации) идентификационный номер (идентификатор), присвоенный признанным банком данных (например, MycoBank). Приводятся предварительные тексты новых статей *Кодекса*, касающихся электронной публикации и предложены наилучшие пути их практического применения.

С тем, чтобы ускорить распространение информации об изменениях, сделанных в *Международном Кодексе Номенклатуры водорослей, грибов и растений*, эта статья будет напечатана в журналах: *BMC Evolutionary Biology, Botanical Journal of the Linnean Society, Brittonia, Cladistics, MycoKeys, Mycotaxon, New Phytologist, North American Fungi, Novon, Opuscula Philolichenum, PhytoKeys, Phytoneuron, Phytotaxa, Plant Diversity and Resources, Systematic Botany* и *Taxon*.

## Введение

На XVIII Международном Ботаническом Конгрессе в Мельбурне, Австралии, в июле 2011 года, были сделаны два важных изменения в *Международном Кодексе Ботанической Номенклатуры *(теперь – *Международный Кодекс Номенклатуры водорослей, грибов и растений*), которые начнут действовать с 1 января 2012 г..Эти изменения будут касаться всех, кто публикует названия соответственно этому *Кодексу*. Поскольку *Кодекс*, принятый в Мельбурне, не будет опубликован примерно до середины 2012 г., мы полагаем, что было бы полезно описать эти изменеия, в частности те, которые касаются эффективной публикации с помощью электронных средств массовой информации (в статьях 29, 30 и 31). Краткий отчет обо всех изменениях, принятых в Мельбурне, см. в McNeill et al. (2011).

Предварительные тексты измененных Статей, Примечаний и Советов *Кодекса* об эффективном обнародовании приведены с тем, чтобы помочь редакторам и издателям разработать наилучшие пути применения *Кодекса* в этом аспекте. Мы также разъясняем здесь, *что* эти изменения *не* означают, чтобы помочь тем, кто хочет обнародовать новые названия и типификации в электронном виде. Мы настоятельно советуем читателям сверяться с отчетом Специального Комитета по Электронным Публикациям (Chapman et al. 2010), где объясняются причины изменений, предложенных перед Конгрессом, и принятых теперь в *Кодексе*.

## Предварительный текст измененных Статей 29, 30 и 31 и Советов 29A, 30A и 31A

Здесь мы приводим текст всех относящихся к случаю Статей, Примечаний и Советов (пропуская Примеры) с изменениями, выделенными **жирным шрифтом**. Предложенный текст – предварительный, в ожидании заседания Редакционного Комитета в декабре 2011 г. и утверждения копии для печати *Мельбурнского Кодекса*.

### Статья 29

*29.1.* Эффективное обнародование осуществляется, согласно настоящему *Кодексу*, через распространение печатного материала (посредством продажи, обмена или дарения) среди широкой публики или, по крайней мере, в ботанические учреждения с доступными для ботаников библиотеками. **Эффективное обнародование осуществляется также посредством распространения материала в электронном виде в формате PDF (см. также Ст. 29.3 и Сов. 29A.1), опубликованном онлайн с Международным Стандартным Серийным Номером (ISSN) или с Международным Стандартным Книжным Номером (ISBN).** Эффективное обнародование не производится путем сообщения новых названий на публичных заседаниях, указанием названий в открытых для публики коллекциях или садах, путем выпуска микрофильмов, сделанных с рукописей, машинописных текстов или других неопубликованных материалов, а также распространением названий на электронных носителях **иным путем, чем описано выше**.

***29.2.* Для целей этой статьи, термин «онлайн» определен как электронно доступный через Международную Мировую Сеть - Интернет (World Wide Web).**

***29.3.* В случае изменения формата PDF принимается последующий международный стандартный формат, сообщенный Генеральным Комитетом (см. Разд. III).**

***29.4.* Содержание конкретной электронной публикации не должно исправляться после того, как она была впервые обнародована. Любые поправки не являются эффективно обнародованными. Для эффективного обнародования исправления или изменения должны быть опубликованы отдельно.**

### Совет 29A

[Существующий Совет заменен следующим:]

***29A.1.* Электронная публикация в формате PDF должна быть совместима со стандартом для архивирования PDF/A (ISO 19005).**

***29A.2.* Авторы должны отдавать предпочтение обнародованию в публикациях, которые архивируются соответственно следующим критериям настолько, насколько возможно (см. также Сов. 29A.1):**

(a)** Материал должен быть размещен в нескольких заслуживающих доверия электронных онлайн-банках данных, например, ISO-сертифицированный репозиторий;**

(b)** Цифровые банки информации должны быть размещены в более чем одном регионе и, предпочтительно, на разных континентах;**

(c)** Также рекомендуется рассылка печатных копий в библиотеки в более чем один регион мира и, предпочтительно, на разные континенты.**

### Статья 30

***30.1.* Обнародование, осуществляемое посредством распространения электронных материалов до 1 января 2012 г., не является эффективным.**

***30.2.* Обнародование не является эффективным, если существуют факты, связанные с публикацией или содержанием публикации, свидетельствующие тому, что эта публикация является только предварительной версией, которая была или будет заменена версией, которую издатель считает окончательной; в этом случае только окончательная версия является эффективно обнародованной.**

*30.3*. Обнародование до 1 января 1953 г. посредством несмываемой автографии является эффективным. Несмываемая автография, произведенная позднее этой даты не является эффективно обнародованной.

*30.4.* Применительно к настоящей статье, несмываемой автографией является рукописный материал, воспроизведенный каким-либо механическим или графическим способом (таким как литография, офсет или гравирование на металле).

*30.5.* Начиная с 1 января 1953 г., обнародование в торговых каталогах или в ненаучных информационных изданиях, а с 1 января 1973 г. и в обменных списках семян, не является эффективным обнародованием.

*30.6.* Начиная с 1 января 1953 г., распространение печатного материала, сопровождающего эксикаты (exsiccatae), не является эффективным обнародованием.

*Примечание 1.* Если печатный материал распространяется также и отдельно от эксикат, это является эффективным обнародованием.

*30.7.* Начиная с 1 января 1953 г., публикация отдельной несериальной работы с указанием, что она является диссертацией, представленной университету или другому образовательному учреждению с целью получения ученой степени, не является эффективным обнародованием, если в самой работе не содержится ясного указания (со ссылкой на требования *Кодекса* к эффективному обнародованию) или другого внутреннего (содержащегося в самой работе – *Прим*. *пер*.) свидетельства, что эта публикация предназначается для эффективного обнародование ее автором или издателем.

*Примечание 2.* Наличие ISBN (International Standard Book Number) или указание названия типографии, издательства или распространителя в оригинальной печатной версии рассматривается как внутреннее свидетельство того, что работа была предназначена для эффективного обнародования.

### Совет 30A

***30A.1.* Предварительная и окончательная версии той же самой электронной публикации должны быть ясно обозначены как таковые при их первом издании.**

*30A.2.* Авторам настоятельно рекомендуется избегать обнародования новых названий и описаний или диагнозов новых таксонов (номенклатурных новаций) в недолговечных печатных материалах любого рода, в частности, в таких, которые воспроизводятся в ограниченном и неопределенном количестве, в которых долговечность текста может быть ограниченной, для которых эффективное обнародование, исходя из количества экземпляров, не является очевидным, или в таких материалах, которые, скорее всего, не дойдут до широкой публики. Авторы должны также избегать обнародования новых названий и описаний или диагнозов в популярной периодике, реферативных журналах или в списках исправлений, прилагаемых к изданиям.

*30A.3.* Для того, чтобы обеспечить доступность во времени и пространстве, авторы, обнародующие номенклатурные новации, должны отдавать предпочтение периодическим изданиям, которые регулярно публикуют таксономические статьи. **В противном случае, копия публикации (опубликованной в печатном или электронном варианте) должна быть послана в центр индексации по соответствующей таксономической группе, а публикации, существующие только в печатном варианте,** должны быть помещены, по крайней мере, в десять, но предпочтительно большее число ботанических или других общедоступных библиотек во всем мире.

*30A.4.* Авторам и редакторам рекомендуется сообщать о номенклатурных новациях в резюме, реферате или перечислять их в указателе публикации.

### Статья 31

*31.1.* Датой эффективного обнародования является дата, в которую печатный или **электронный** материал стал доступным, как определено в Ст. 29 и 30. При отсутствии доказательств, устанавливающих какую-либо другую дату, правильной следует считать дату, указанную на печатной или **электронной** копии.

[Существующее Примечание 1 заменено следующим:]

***31.2.* Если обнародование осуществлено параллельным выпуском электронной и печатной версий, это является эффективным обнародованием с одной и той же датой, если даты на копиях не разные, соответственно Ст. 31.1.**

*31.3.* Если отдельные оттиски из периодических изданий или других работ, поступающих в продажу, выпускаются раньше этих публикаций, то дата оттиска принимается как дата эффективного обнародования, если нет доказательств, что она ошибочна.

### Совет 31A

*31A.1.* За дату эффективного обнародования печатного произведения следует принимать ту дату, в которую издатель или его представитель выпускает это произведение в свет одним из обычных способов.

## Рекомендации

Авторы новых названий, редакторы и издатели будут заинтересованы в обеспечении того, чтобы публикации, включающие новые названия, соответствовали *Мельбурнскому Кодексу* и чтобы названия в них были эффективно обнародованы. Мы предлагаем авторам публикаций в журналах или серийных монографиях и книгах, издаваемых онлайн, обмениваться информацией с редакторами, чтобы, как можно быстрее, внедрить лучшие методы в научном сообществе. В течение некоторого времени многие издатели внимательно адресовали проблемы, связанные с электронной публикацией новаций (см. Knapp and Wright 2010; руководство в PLoS One [http://www.plosone.org/static/policies.action#taxon]), и значительный интерес к внедрению этих изменений в *Кодекс *был очевидным.

Некоторые рекомендации, которые, мы надеемся, смогут помочь на начальной стадии электронной публикации новаций соответственно *Мельбурнскому Кодексу* приведены ниже:

• Рекомендуется выделять в каждой статье даты публикации (как это сделано в большинстве журналов, например, *New Phytologist* или *Nature*).
• Если более ранний выпуск онлайн-версии не является таким же, как окончательная версия (и поэтому не является эффективно обнародованным), рекомендуется отметить этот факт штампом (пример - *American Journal of Botany*).
• Включение и выделение ISSN или ISBN публикации в каждой статье поможет регистрирующему персоналу установить, эффективна ли публикация.
• Публикации в журналах (или серийных монографиях), участвующих в системе CLOCKSS (см. описание Knapp and Wright 2010) или в других международных архивах и системах хранения данных, обеспечат долгосрочное хранение.
• Авторы электронно обнародуемых новых названий должны уведомлять соответствующие центры индексации, как рекомендовано в Сов.30.А.3 – это поможет регистрирующему персоналу, который, иначе, может не знать об электронно обнародованных названиях.

## Что эти изменения *не* означают

Не смотря на то, что новые Статьи и Рекомендации *Кодекса* используют термины PDF и PDF/A, это не означает, что публикации должны быть изданы *только *в этом формате с тем, чтобы быть эффективно опубликованными. Например, некоторые онлайн-журналы издаются параллельно в формате HTML (Hypertext Markup Language) вместе с PDF версией. В таких случаях PDF версия будет эффективно опубликована. Соглашение о том, что Генеральный Комитет по Ботанической Номенклатуре будет сообщать о новом приемлемом международном стандартном формате, в случае, если PDF будет заменен, означает, что авторы новаций и научная общественность, ипользующие *Кодекс,* будут информированы о новшествах в этой области, и *Кодекс* будет защищен от устаревания.

Новации, опубликованные ниже перечисленными способами *не* будут эффективно обнародованы согласно *Мельнбурнскому Кодексу*:

• Публикации на веб-сайтах или в недолговечных документах, доступных в Интернет (существуют строгие критерии получения ISSN [http://www.issn.org]).
• Публикации в журналах без зарегистрированного ISSN или e-ISSN.
• Публикации в книгах без зарегистрированного ISВN или e-ISВN.

Совет о том, что печатную копию каждой электронной публикации следует помещать в библиотеку рекомендует ботаникам, как поступать, но не устанавливает стандарты или протокол действий для сотрудников библиотек. Библиотекари находятся в сложном переходном состоянии между разными методами публикации (Johnson and Luther 2007), и, в случае большого объема присылаемого материала, ботаники могут оказаться в ситуации, когда работники библиотек откажутся принять печатные оттиски отдельных статей.

## Два других важных изменения в *Кодексе*, относящихся к обнародованию названий

Второе изменение в *Кодексе*, утвержденное в Мельбурне и вступающее в действие с 1 января 2012 г. гласит, что описание или диагноз, требуемые для действительного обнародования названия нового таксона для всех организмов, попадающих под действие *Кодекса*, могут быть опубликованы на любом из двух языков, английском, либо латинском. В настоящее время это положение действует для ископаемых растений, но для всех неископаемых таксонов требуются латинские описание или диагноз (грибы и растения с 1 января 1958; водоросли [включая цианобактерии, попадающие под действие *Кодекса*] с 1 января 1958 г.). Это не относится к форме научных названий, которые остаются латинскими или принимаются как латинские. Требования отдельных журналов относительно латинского или английского языков, будет определяться, конечно же, редакторами этих журналов.

Третье изменение в *Кодексе*, утвержденное в Мельбурне и относящееся к обнародованию названий, но не вступающее в действие до 1 января 2013 г. (a не 1 января 2012 г. как ошибочно отмечено Миллером и др. (Miller et al. 2011)) гласит, что для всех новых названий организмов, понимаемых как грибы, в качестве дополнительного требования для действительного обнародования необходимо цитировать в протологе (все, что связано с названием и его дествительным обнародованием) идентификационный номер, выданный международно признанным банком данных (таким, как MycoBank [http://www.mycobank.org/]). Об этом пойдет речь в отдельной публикации.

Требование наличия уникального идентификатора для новых названий грибов, начиная с 1 января 2013 г., *не* касается растений и водорослей; авторам новых названий для этих групп не нужно запрашивать LSIDs (Life Science Identifiers) или другие идентификаторы в центрах по индексации.
